# Discrete Emotion Effects on Lexical Decision Response Times

**DOI:** 10.1371/journal.pone.0023743

**Published:** 2011-08-24

**Authors:** Benny B. Briesemeister, Lars Kuchinke, Arthur M. Jacobs

**Affiliations:** 1 Allgemeine und Neurokognitive Psychologie, Free University Berlin, Berlin, Germany; 2 Department of Psychology, Ruhr University Bochum, Bochum, Germany; 3 Dahlem Institute for Neuroimaging of Emotion (D.I.N.E.), The Cluster of Excellence “Languages of Emotion”, Free University Berlin, Berlin, Germany; French National Centre for Scientific Research, France

## Abstract

Our knowledge about affective processes, especially concerning effects on cognitive demands like word processing, is increasing steadily. Several studies consistently document valence and arousal effects, and although there is some debate on possible interactions and different notions of valence, broad agreement on a two dimensional model of affective space has been achieved. Alternative models like the discrete emotion theory have received little interest in word recognition research so far. Using backward elimination and multiple regression analyses, we show that five discrete emotions (i.e., happiness, disgust, fear, anger and sadness) explain as much variance as two published dimensional models assuming continuous or categorical valence, with the variables happiness, disgust and fear significantly contributing to this account. Moreover, these effects even persist in an experiment with discrete emotion conditions when the stimuli are controlled for emotional valence and arousal levels. We interpret this result as evidence for discrete emotion effects in visual word recognition that cannot be explained by the two dimensional affective space account.

## Experiment 1

### Introduction

Single word recognition, that is the mechanisms of identifying the meaning of a written or spoken word, is standardly investigated by means of the lexical decision task (LDT), where participants judge the lexical status of a presented letter string on whether it is a correct word (*e.g.* ‘PAPER'), or not (pseudo- or nonwords, *e.g.* ‘PAPET'). Given that cognitive and affective processes highly interact, it is not surprising that psycholinguistic research revealed effects of affective variables in word recognition by manipulating the emotionality of the presented words [1]–[9]. These experimental manipulations are often operationalized along the two dimensions of the affective space, namely emotional valence, which indicates whether a stimulus is positive or negative, and emotional arousal, which describes the emotional intensity associated with the stimulus that can be linked to physiological activation [10]–[13].

Both, effects of emotional valence and arousal on word processing are well documented. While positive valence is known to facilitate lexical processing in the LDT [2], [4], [5], [8], [9], a facilitatory effect for negatively valenced words is observed only at high levels of emotional arousal [1], [2], [6], [7]. At low arousal levels, negative stimuli are sometimes processed even slower than comparable neutral words [1], [6].

Concerning the valence effects, two theoretically distinct explanations dominate the literature on emotional word recognition. A first explanation is based on the view that emotions emerge from two underlying motivational systems, appetitive and aversive [3], [14], [15]. According to this view, highly valenced stimuli lead to faster approach or avoidance responses than less valenced stimuli and therefore to differences in processing speed. Valence is considered a continuous dimension in these approaches, with a stepless transition from the positive to the negative pole and a neutral midpoint. Estes and Adelman [16], [17], in contrast, derived their categorical valence conception from the automatic vigilance [18] and automatic affective evaluation [19] models, which state that all stimuli are evaluated automatically on their affective value as either being positive (appetitive) or negative (aversive). In this conception, emotional stimuli vary more between the affective categories than within [17] . According to Estes and Adelman a further differentiation within the positive category and within the negative category is not reasonable. Both theories are supported by experimental evidence (for continuous valence, see [3], [6]; for categorical valence, see [16], [17], [20]–[22]).

As a consequence, Estes and Adelman correctly predict that response times (RTs) in visual word recognition vary with emotional categories, but not as a function of emotional intensity within the positive or negative category [16], [17]. Moreover, they are able to show that their model explains a comparable amount of variance as the continuous model [6] in a multiple regression analysis on lexical decision performance data, while being more parsimonious in terms of the models' explanatory value due to five fewer explanatory variables. Still, criticism was raised regarding the appropriateness of the database used in Estes & Adelman [16], [17]. Kousta et al. [3] discussed that the valence norms in Estes & Adelman are not normally distributed which might bias the results of the regression analyses reported therein, and that the amount of neutral words was underrepresented in this study. Accordingly, relying on a larger corpus with more neutral stimuli Kousta et al. [3] again found evidence in support of the continuous valence conception (but didn't directly contrast the two accounts).

Models relying on emotional valence and arousal are the most dominant models in the literature on emotional processing, but they are not without alternatives. From an evolutionary view, it is often assumed that human emotions are categorized in terms of discrete emotions [23]–[26]. Unlike the continuous valence model, discrete emotion theories suggest discrete emotion categories. And unlike the categorical valence model, it is suggested that both, positive and negative valence category are further differentiated. At least five different discrete emotion categories – happiness, sadness, anger, fear, disgust – can be identified from facial or vocal expression. This ability to discriminate biologically significant expressions is discussed as an inborn ability and has been shown to generalize across different human cultures. Besides their origin in biological markers, discrete emotions are also elicited by other types of ecological valid stimuli, such as film clips [27], [28], complex pictures [29] and verbal descriptions [30]–[32].

An evolutionary explanation is not plausible for these stimulus types, but contextual learning has been suggested as a key process in linking such stimulus material to discrete emotions [31]. Emotion categories acquired during childhood may facilitate the perception of discrete emotions in different circumstances, a mechanism that is most probably moderated by the use of language [31] which itself is known to be closely linked to phylogenetically old brain systems responsible for emotional processes [33]. Accordingly, it seems plausible to assume that single word stimuli are also linked to discrete emotion categories. First evidence already documents that discrete emotion data affect lexical decision performance in clinical [34], [35] and non-clinical populations [36].

The present study was designed to further examine the role of discrete emotion categories in visual word recognition and to contrast these data with the predictions of continuous and categorical models of the affective space. In the first step, an automatic selection procedure was computed to reveal the best predicting affective variables for lexical decision RTs derived from a large corpus of lexical decision data. These were then validated using multiple regression analyses in a second step. Analyses were computed using the ANEW database [10] and the ANEW discrete emotion extension by Stevenson, Mikels and James [37] to predict normative lexical decision human performance data provided by the English Lexicon Project database (ELP, [38]). The ANEW contains normative valence and arousal rating data for more than 1000 English words, which has been extended to also account for normative discrete emotion rating data for happy, anger, sad, fear and disgust discrete emotion categories by [38]. The ELP was chosen as the dependent variable because it contains lexical decision performances from more than 800 subjects on more than 40.000 words. This data was collected across six universities, and has become a standard tool for the investigation of lexical processing [6], [16], [17], thus allowing for a maximum reproducibility. The results of our analyses suggest that discrete emotion information has a comparable or even enhanced explanatory value as the continuous and the categorical model. To further verify these results on independent data and to overcome the problems of the ANEW database [3], a final lexical decision experiment comprising a factorial variation of discrete emotion content while controlling for effects of valence and arousal was conducted to replicate the multiple regression results.

#### Backward elimination

Automatic selection procedures are a good possibility to statistically explore which predictors explain most variance in a dependent variable (for details, see [39]). Reisenzein [32] documented a close relationship between discrete emotion labels and the dimensional affective space model by showing that discrete emotion words show stable patterns across different intensities along the valence-arousal dimensions. Thus, all three models, the continuous valence model, the categorical valence model and the discrete emotion model, are likely to share considerable variance, which can cause the problem of multicollinearity. Automatic selection procedures in multiple regression analyses avoid multicollinearity, and help to identify the variables that individually account for a significant amount of variance.

Searching for the most promising predictors, we presented affective variables from all three models to the automatic selection procedure, together with other psycholinguistic predictors known to affect lexical decisions (*e.g.*, stimulus length and frequency, see [3], [6], [40], using the average lexical decision times taken from the ELP as the dependent variable. Because of the very univocal literature, valence and arousal were expected to explain reliable variance in the human performance data. Finding discrete emotion variables among the selected variables would, however, strongly support the hypothesis of discrete emotion influences on single word processing.

### Materials and Methods

To obtain a data set for the subsequent regression analyses, we followed the procedure described by Estes and Adelman [16], [17] and Larsen et al. [6]. Stimulus data from ANEW [10] was merged with lexical decision RTs collected from the ELP [37]. The ELP has collected the performance data in a standardized lexical decision implementation: 40,481 words and 40,481 nonwords were presented to 816 native English subjects in uppercase QBASIC font letters. Each trial began with the presentation of three asterisks for 250 ms, followed by a 50 ms tone and a blank screen for 250 ms. Stimuli remained on screen until button press or for 4 seconds, whichever occurred first. The next trial started after a fixed inter-stimulus-interval of 1,000 ms, and behavioral errors were reported back to the subject.

In addition to the ELP and ANEW data, we added English discrete emotion norms to the data set, collected and published by Stevenson et al. [38] for the ANEW. This resulted in a list of 1.023 words. A total of 14 variables was used for backward elimination, namely the psycholinguistic variables logarithm of HAL frequency [41], stimulus length [42], orthographic neighborhood size [43]–[45], syllables, mean bigram frequency [46], plus the following affective variables: The continuous model variables' continuous valence, arousal and their first-order interaction, the categorical model variable categorical valence, with ANEW valence greater than 5 assigned to positive and ANEW valence smaller than 5 assigned to negative category (definition taken from [16]; the word 'TAXI', having ANEW valence of 5, was excluded, leaving 1022 words for analysis), and the discrete emotion variables happiness, anger, fear, disgust and sadness [38]. All variables were centered, and entered in a second step into a multiple regression analysis, using RT as the dependent variable. A backward elimination procedure was applied using SPSS software (version 13.0, SPSS Inc., USA), with standard p-to-leave of 0.1.

### Results

An overview of the selection results including the estimated betas is given in Table 1. Six variables survived the backward elimination procedure, among them the three discrete emotions variables happiness, fear and disgust. No other affective variable survived. The valence*arousal interaction was eliminated as first affective predictor at second position, categorical valence as last (see Table 1). As expected, frequency and length were the best predictors.

**Table 1:** Backward elimination results.

Step	Variable	beta	t-value	p-value
1. removal	bigram frequency	-0.002	-0.067	0.947
2. removal	valence*arousal	-0.003	-0.106	0.915
3. removal	anger	-0.007	-0.148	0.883
4. removal	sadness	-0.014	-0.305	0.760
5. removal	arousal	-0.026	-0.888	0.375
6. removal	N	0.031	1.082	0.280
7. removal	dimensional valence	0.084	1.087	0.277
8. removal	categorical valence	-0.055	-1.318	0.188
**9. final model**	log HAL frequency	-0.469	-18.791	<0.001
	length	0.261	7.565	<0.001
	syllables	0.131	3.950	<0.001
	happiness	-0.091	-2.983	0.003
	disgust	0.089	2.948	0.003
	fear	-0.083	-2.721	0.007

**Note**: N = orthographic neighborhood size.

## Experiment 2

The automatic selection results show a consistent picture in favour of a discrete emotion explanation of lexical decision times. Neither continuous valence, as expected according to Larsen et al. [6] or Kousta et al. [3] for example, nor categorical valence as expected according to Estes and Adelman [16], [17], nor emotional arousal or the valence*arousal interaction were identified as predictive affective variables, but three out of five discrete emotion variables, suggesting that happiness, fear and disgust explain significant variance in human RTs. This analysis clearly documents that discrete emotions predict word processing performance in healthy subjects [36].

Still, these results should be interpreted with caution. Automatic selection procedures select the variables that individually account for most variance, but they do not necessarily identify the best theoretically reasonable model. A final conclusion concerning the predictive power of the three emotion models discussed above is not possible on the basis of this analysis alone. In fact, it is quite likely that dimensional models, which claim to account for the entire affective space [32], perform much better than a model including only a limited number of discrete emotions, each of which is by definition limited in explanatory value.

To directly compare the predictive power of the continuous valence model as published by Larsen et al. [6] and the categorical model published by Estes and Adelman [16] with a model including five discrete emotions (*i.e.*, happiness, anger, fear, disgust and sadness), a multiple regression analysis was conducted. Again, best overall performance would be expected from the categorical model [16], [17] or the continuous model [3], [6], considering the literature. Given the automatic selection results and the behavioral relevance of discrete emotions, however, we expected the discrete emotion model to perform at least comparably well.

### Materials and Methods

Again, the ELP, the ANEW and the discrete emotion data from Stevenson et al. [38] were merged. All three models were used to predict standardized RTs with centered variables, following Larsen et al. [6]. As suggested in Larsen et al. [6], the continuous model contained the predictors length, log HAL frequency, orthographic neighborhood size, syllables, valence, arousal, squared valence, valence by arousal interaction, cubed valence, squared valence by arousal interaction and cubed valence by arousal interaction. The categorical model, following Estes and Adelman [16], predicted RTs with the variables length, log HAL frequency, orthographic neighborhood size, syllables, arousal and categorical valence. Contextual diversity was included, which however does not significantly affect overall performance of the regression model as published by Estes and Adelman [16]. Finally, in the discrete emotion model, length, log HAL frequency, orthographic neighborhood size, syllables, and the five discrete emotion variables happiness, anger, fear, disgust and sadness were used to predict RTs. Except for the affective variables, all three regressions used the same predictors. Although the original continuous model from Larsen et al. [6] did not contain syllables as predictor, it was added in this analysis to ease interpretation of the results. Linear multiple regressions were calculated using SPSS software, level of significance was set to 0.05.

### Results

The continuous regression model altogether accounted for 59.0% of the variance (adjusted R square), with length, log HAL frequency, syllables, valence, valence by arousal interaction, cubed valence and cubed valence by arousal interaction as significant predictors. Overall model performance differs from Larsen et al. [6] because we did not use hierarchical regression analysis, which overestimates predictive power. The categorical regression model explained a total of 58.7% variance (adjusted R square) with length, log HAL frequency, syllables, categorical valence and arousal as significant predictors. The discrete emotion model, finally, with significant predictors length, log HAL frequency, syllables, happiness, fear and disgust, accounted for 59.6% variance in RTs (adjusted R square). All three models are summarized in Table 2.

**Table 2:** Comparison of three affective regression models.

Variable	Categorical model			Continuous model			Discrete emotion model		
	beta	t-value	p-value	beta	t-value	p-value	beta	t-value	p-value
Log HAL	-0.505	-21.477	<0.001	-0.501	-21.408	<0.001	-0.482	-20.423	<0.001
Length	0.294	8.308	<0.001	0.301	8.525	<0.001	0.316	8.983	<0.001
Syllables	0.131	4.129	<0.001	0.125	3.961	<0.001	0.131	4.160	<0.001
N	0.041	1.475	0.140	0.043	1.555	0.120	0.045	1.661	0.097
Val (cat)	-0.101	-4.650	<0.001						
arous	-0.046	-2.207	0.028	-0.009	-0.250	0.802			
Val (con)				-0.201	-3.820	<0.001			
Val*arous				0.197	3.496	<0.001			
Val^2^				-0.028	-1.079	0.281			
Val^2^*arous				-0.020	-0.581	0.561			
Val^3^				0.127	2.156	0.031			
Val^3^*arous				-0.190	-3.066	0.002			
Happiness							-0.114	-3.818	<0.001
Disgust							0.137	4.542	<0.001
Fear							-0.075	-2.018	0.044
Sadness							-0.025	-0.658	0.511
Anger							-0.046	-1.185	0.236
**Adj. R^2^**	0.587			0.590			0.596		

**Note**: Log HAL  =  logarithm of HAL frequency, N = orthographical neighborhood size, Val (cat)  =  categorical valence, Val (con)/Val  =  continuous valence, arous  =  arousal

### Discussion

Three affective variables signaling the amount of happiness, fear and disgust significantly predict lexical decision RTs according to the automatic selection procedure. When comparing the overall performance of a regression model with five discrete emotion variables with those of categorical and continuous models discussed in the literature [6], [16], all three perform more or less equally well. This is not trivial, since dimensional models often claim to account for the entire affective space, while discrete emotions, by definition, are more specific [47]. The multiple regression analysis, however, documents that five discrete emotions explain just as much (or even slightly more) variance as both, the dimensional and the categorical model.

The overall RT pattern known from the experimental visual word recognition literature was replicated [1], [4], [5], [7]. Positive valence is consistently accompanied by faster RTs, whereas negative words show indifferent results with sometimes increased and sometimes decreased RTs as compared to neutral words [1], [5]. According to the above regression analyses, negative betas for valence and arousal indicate that the dimensional and the categorical model both predict that positive stimuli are processed faster than negative stimuli and that high arousal facilitates processing. The dimensional and the categorical model only differ in their expectations for within valence effects, which is discussed excellently and in great detail in Estes and Adelman [17].

**Figure 1:**
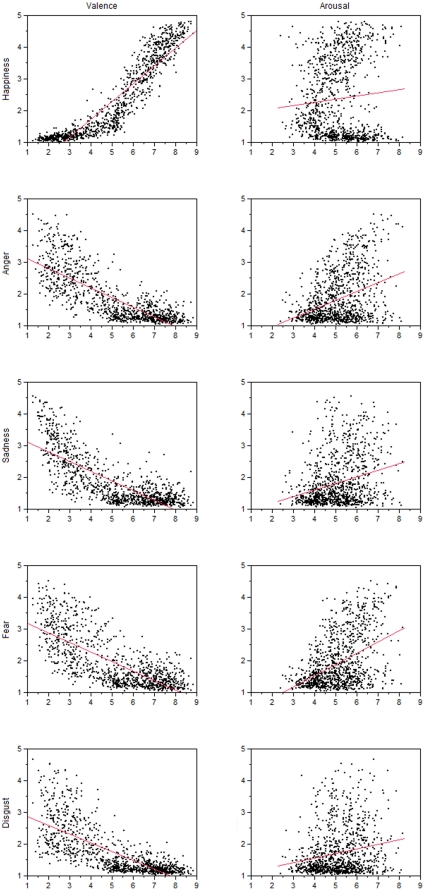
The relationship between the five discrete emotion variables happiness, anger, sadness, fear and disgust and the two affective space variables valence (left column) and arousal (right column).

Concerning discrete emotions, the regression model predicts faster RTs with increasing values of happiness and fear, and slower RTs when disgust levels increase. Happiness related words (*i.e.*, positive words) are predicted to elicit faster RTs, whereas negative words would show indifferent results depending on the proportion of fear and disgust-related words in the stimulus set. Following the predictions of the discrete emotion model, the proportions of the different negatively valenced discrete emotion words in a given data set explain the indifferent results regarding negative words. So far, the two dimensional affective space models and the discrete emotion model basically predict the RT pattern. Considering the bivariate relationships between the valence and arousal norms from the ANEW database and the discrete emotion norms, there is an interesting and crucial difference between the models, however. According to Stevenson, Mikels and James [48] and as visible in Figure 1, all discrete emotion variables are positively related to emotional arousal, even disgust. Higher levels of disgust are therefore related not only to higher negativity, but also to higher arousal (see Figure 1 and [48]). This can explain why arousal did not account for a significant proportion of RT variance under the discrete emotion model. Moreover it challenges the two dimensional approaches which both predict that highly arousing negative stimuli are processed faster instead of being processed more slowly, as expected from the discrete emotion models' regression data.

## Experiment 3

In order to directly test the predictions of the regression model on discrete emotions, an additional experiment was designed. Following the above analyses one would expect faster RTs to both happiness and fear-related words and slower responses to disgust-related words in a LDT. Since the backward elimination regression did not reveal effects of valence or arousal, we predict that discrete emotion effects are still observed even when the stimulus material is controlled for levels of valence and arousal (according to the dimensional affective space model). Five stimulus conditions were created containing words which, according to the discrete emotion model, are related to either happiness, disgust, fear, anger, or no other discrete emotion (*i.e.*, neutral). The neutral condition consisted of words that show overall low levels of discrete emotion intensities. Sadness was not included as a further condition in the experiment because the German database that provides the discrete emotion norms does not contain sufficient sadness related stimuli to fulfill the high matching standards used in this study. Still, based on the regressions analyses presented above one would not have predicted sadness related effects on the lexical decision performance data. Across all conditions, arousal was carefully controlled, and as an additional constraint, the three negative conditions did not differ in valence. Both the dimensional and the categorical model predict a valence effect with faster responses to happiness related words, intermediate responses to neutral and slowest responses to negative words (at intermediate levels of arousal). Since all three negative discrete emotion conditions have similar levels of valence and arousal, the dimensional models would not predict RT differences between them. In contrast, we expected to find strong discrete emotion influences on word processing. Following the direction of the respective beta values from the regression analysis, we predict to observe slowed-down processing of disgust-related and speeded processing of happiness related words, with RTs to anger and fear-related words lying in between. Even between the latter two discrete emotion conditions a slight processing advantage for fear-related words could be predicted based on differences in the respective betas in Table 2.

### Materials and Methods

#### Ethics

The authors took care that this study was conducted in accordance with the declaration of Helsinki and under the ethical guidelines of the German science foundation, although the study was not presented to and therefore not approved by any ethical committee or institutional board. Since the lexical decision paradigm is a standard paradigm in psycholinguistic research that involves no harm to the subjects, collects no personally critical information and has a long history in psycholinguistic research, a specific approval for this study was considered not necessary by the authors. All subjects were informed prior to their inclusion in the study on their right to decline to participate and to abort the experiment without consequences, and they were informed about the goals of the study. All participants gave their informed consent verbally prior to their inclusion. Written consent was not considered to be necessary by the authors since verbal consent already is a legal contract according to the German law. The authors alone are responsible for any decision concerning the ethics of this study.

#### Participants

A total of 21 native German subjects (19 female; 19 right handed, 1 reporting to be ambidextrous; mean age  = 25.4, S.D. = 6.6, range  = 19 to 42), recruited at the Freie Universität Berlin, participated in this study. Some of them received course credit for participation, others participated without recompense.

#### Materials

Stimulus material consisted of 125 nouns taken from the Discrete Emotion Norms for Nouns – a Berlin Affective Word List (DENN-BAWL, [36]) and an equal number of nonwords. Within the word set, five conditions (happiness, neutral, fear, anger, disgust) were constructed, each containing 25 items of 4–6 letters length. Words defined as being neutral for this study had valence ratings lying between −0.5 and +0.5 according to the Berlin Affective Word List Reloaded [49] and low discrete emotion intensities (mean discrete emotion ratings below 2.25). ‘Positive' words had a valence rating above 1 and their happiness rating was higher than their respective rating in any other discrete emotion category. ‘Negative' words, finally, had a valence rating below -1. Words in disgust condition had higher disgust than fear, sadness or anger values, equivalent relations were used to define fear and anger conditions.

All five conditions were matched on arousal [mean arousal (and SD) for happiness  = 3.4 (0.5); for fear  = 3.4 (0.4); for anger  = 3.4 (0.6); for disgust  = 3.2 (0.4); for neutral  = 3.3 (0.4)] as well as their number of letters, syllables, phonemes and orthographical neighbors, their frequency, their imageability and their averaged bigram frequency using an ANOVA (F<1). Additionally, the three negative basic emotion conditions were matched on valence [mean valence (and SD) for anger  = −1.5 (0.4); for fear  = −1.6 (0.4); for disgust  = −1.6 (0.4), F<1; mean valence (and SD) for happiness  = 1.9 (0.5); for neutral  = 0.0 (0.3)]. Estimates were taken from the BAWL-R.

Nonwords were created by selecting an additional 125 words of 4–6 letters length from the BAWL-R and replacing one or two letters, vowels with vowels and consonants with consonants, thus creating pronounceable but meaningless letter strings. They did not differ from words in length and number of syllables in a t-test (t<1).

#### Procedure and data preparation

Participants were seated in a quiet room in front of a 15 in. laptop screen. They were instructed to decide as fast and as accurate as possible whether a presented letter string is a correct German word or a nonword. The decision was made using left and right index finger, lying on the SHIFT buttons. The button-to-response assignment was counterbalanced across subjects. After nine practice trials not part of the stimulus set and therefore excluded from any analysis, the experimenter left the room, provided that subjects did not have further questions.

Stimuli were presented by Presentation 9.9 software (Neurobehavioral Systems Inc., Canada) in randomized order in the center of the screen, written in black uppercase letters (font type “Arial”, size 24) on a blank white screen. Each trial began with a fixation cross (+) presented for 500ms in the center of the screen, replaced by the stimulus (500 ms) and another fixation cross, presented until button press.

For analyses, error-free mean RTs were calculated for each condition and each participant. Outliers (3.7%), defined as responses faster or slower than the individual mean RT±2 S.D., were excluded from analysis. For error analyses, behavioral errors were summed up per participant and condition. Subjects committed 7.5% errors on average. One subject was excluded having committed more than 20% behavioral errors. All analyses were computed using SPSS software at an a-priori significance level of 0.05.

### Results

**Figure 2:**
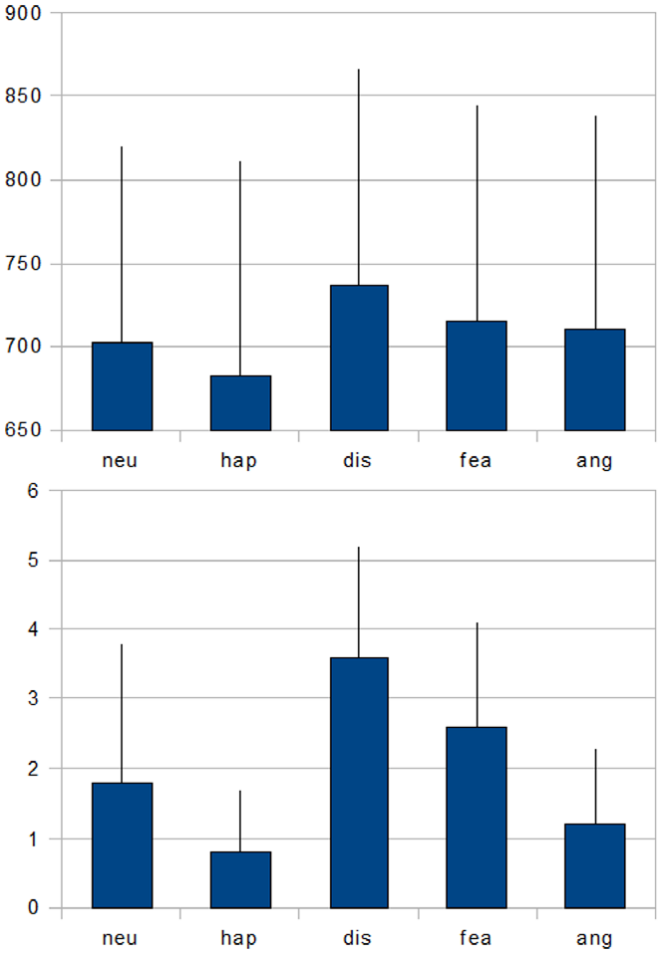
Mean response times in ms (upper part) and summed error rates (lower part) for the lexical decision task. Error bars represent one standard deviation.

A repeated measures ANOVA over all five conditions (happiness, neutral, fear, anger, disgust) revealed a significant discrete emotion effect in RTs [F(4,16)  = 9.072, p<0.001]. Planned pairwise comparisons using matched pairs t-tests revealed faster responses to happiness related words (mean  = 682.6 ms, S.D.  = 128.4 ms) when compared to neutral words (mean  = 702.0 ms, S.D.  = 118.0 ms; t(18)  = 2.625, p  = 0.017). Correct recognition of disgust-related words (mean  = 737.4 ms, S.D.  = 129.9 ms) took significantly longer than recognizing fear (mean  = 714.6 ms, S.D.  = 130.4 ms; t(18)  = −2.349, p  = 0.030) or anger related stimuli (mean  = 710.9 ms, S.D.  = 127.8 ms; t(18) = −2.272, p  = 0.035). All three negative conditions yielded in slower RTs than happiness related words (happiness vs. disgust: t(18) = 5.280, p<0.001; vs. fear: t(18) = 3.973, p = 0.001; vs. anger: t(18)  = 3.242, p = 0.004), but unlike disgust, neither fear nor anger related words differed from neutral stimuli (neutral vs. disgust: t(18) = −3.795, p = 0.001). These results are also depicted in Figure 2.

Analysing the error rates, a repeated measures ANOVA over all five conditions (happiness, neutral, fear, anger, disgust) revealed a significant effect [F(4,15)  = 19.970, p<0.001]. Planned pairwise comparisons using matched pairs t-tests revealed more errors while recognizing disgust-related words (mean sum of errors  = 3.6, S.D.  = 1.6) than in any other condition (disgust vs. neutral: t(18)  = 4.487, p<0.001; vs. fear: t(18)  = 3.012, p  = 0.007; vs. anger: t(18)  = 5.811, p<0.001; vs. happiness: t(18)  = 7.520, p<0.001). Fear-related stimuli (mean sum of errors  = 2.6, S.D.  = 1.5) lead to more errors than anger related (mean sum of errors  = 1.2, S.D.  = 1.1; t(18)  = 4.762, p<0.001), happiness related (mean sum of errors  = 0.8, S.D.  = 0.9; t(18)  = 6.514, p<0.001) and neutral stimuli (mean sum of errors  = 1.8, S.D.  = 2.0; t(18)  = 2.212, p  = 0.040). Happiness and neutral condition differed significantly (t(18) = -2.730, p = 0.013).

### Discussion

Discrete emotion conditions significantly affect subjects RTs and error data in visual word recognition even when the stimuli are controlled for their levels of arousal and valence (the latter within the ‘negative' conditions). As such, the present study supports a discrete emotion model in visual word recognition that incorporates an explanatory value which is superior to the standard two-dimensional affective space model or the categorical valence model. The LDT results resemble the predictions made following the above regression analyses. In an automatic selection procedure, the three discrete emotion categories happiness, fear and disgust were selected as the only affective variables predicting word recognition performance. Neither valence nor arousal explained additional variance. A subsequent linear multiple regression confirmed these predictors, extended by the observation that such a discrete emotion model behaves comparably well and accounts for just as much variance as a dimensional valence-arousal model [3], [6] or a categorical model [16], [17].

Following the criticisms of Kousta et al. [3] in response to Estes and Adelman [16], the final experimental study used German nouns rated for valence, arousal and five discrete emotions to overcome the methodological problems associated with the ANEW data. A processing advantage of happiness related words and a slowed processing of disgust-related words compared with neutral words was observed. Fear-related words could not be differentiated from neutral words in terms of their RTs and also did not show the predicted processing advantage compared with anger-related words. But looking at the error data, it seems that the participants showed a (not predicted) trade-off, when fear-related words led to more errors compared to the neutral and the anger conditions. Probably, this speed-accuracy trade-off could be attributed to differences in the lexical decision paradigm employed here as compared with that of the ELP (*e.g.*, shorter inter-trial intervals, no feedback, shorter stimulus presentation duration), but this explanation needs to be further examined in subsequent studies.

Overall, these results have two immediate implications: First, given the data we were not able to replicate the observed processing advantage of both positive and negative words, as proposed by Kousta et al. [3]. In contrast, our data correspond to earlier findings, showing that processing of negative words is slowed when emotional and neutral words are controlled for their level of arousal [1], [16], [17], which is best explained by a non-linear relationship between negative valence, arousal and RTs (see Figure 2 in [6]). Only high arousal words show the proposed processing advantage, whereas negative valence itself seems to slow RTs. As such, our data support automatic evaluation approaches [16]–[19] that propose a fast processing of stimulus' valence. The contribution of arousal to this process, however, is not clear yet, although first neurophysiological studies indicate that words' arousal may alter early lexico-semantic processing independent of affective evaluation [1], [50], [51].

Secondly and most important, valence and arousal are not sufficient to explain subjects' word recognition performance within negatively valenced words. A simple positive-negative evaluation does not explain the processing differences within negative words with slowed RTs and higher error rates for disgust-related words, nor does it account for the relatively slowed processing and decreased error rates for fear-related words. Thus, neither a continuous valence arousal model of affective space [3], [6] nor a categorical valence model [16] can explain the performance effects within these negative word categories. Additional knowledge of discrete emotion category membership is required to explain the performance differences. Although the processing of negative words is slowed in general, different processes seem to distinguish disgust, fear and anger related words. Disgust words are processed slowest, thus seem to attract most processing resources according to the automatic evaluation hypothesis [18], [19]. In contrast, fear-related words show a relative processing facilitation, indicated by faster and more accurate responding as compared with disgust-related words. In general, we propose contextual learning as suggested by Feldman Barrett et al. [31] and as described in the introduction to explain these effects. The contextual learning hypothesis refers to the assumption that discrete emotion categories acquired during early childhood may facilitate the perception of discrete emotions in different circumstances and that the perception itself is moderated by the use of language (see also [31] for a discussion of the tight link between emotion and language). The data presented here suggests that contextual learning is indeed specific for discrete emotions and less powerfull for the learning of dimensional or bi-modal models.

In sum, with the highly concordant data from different analyses performed in different languages we present strong evidence for the existence of a discrete emotion specificity in visual word recognition. These results can be taken as an indication that the dimensional models or bi-modal categorical models of affective space are underdetermined in explaining human performance in visual word recognition [52]. The results presented here complement a previous study by Stevenson et al. [53], which examined explicit evaluative judgments of emotionally and sexually arousing words on 11 affective variables: the three affective dimensions, five discrete emotion categories and three additional rating of sexual categories. Based on a data-driven factor analysis approach, four independent factors were identified that account for most of the variance in the subjective ratings. Three out of these four factors represent the discrete emotions happiness, disgust, and a basic aversive category (covering both fear and sadness), the fourth factor representing a sexual category. Affective dimensions, in contrast, did not explain much variance in the subjective ratings. Thus, the present results together with the Stevenson et al. [53] study demonstrate the appropriateness of discrete emotion categories in explaining affective rating behavior, and furthermore, with the lexical decision data presented above we are able to show that discrete emotion effects can also be observed in visual word recognition, where the processing of the emotional content is incidental to the task requirements. Of note here is that Stevenson et al. [53] observed sex differences in their rating data, a question that could not be addressed with the present study because of an unbalanced proportion of female and male participants. It remains for future studies to investigate whether sex related differences can be observed within discrete emotion effects on the LDT.

#### Implications for future studies

While the overall performance of dimensional models is comparable to that of a discrete emotion model, we show that a two dimensional perspective - regardless of the specific valence conception [3], [6], [16], [17] - fails to correctly predict discrete emotion effects for negative words in visual word recognition. Still, this paper is no more than a first glimpse on discrete emotion effects on word processing, leading to several implications for future studies. First of all, it would be interesting to see which further discrete emotion variables affect word processing. While sadness ratings are already available in English and in German [36], [38], further discrete emotions have been suggested in the literature (*i.e.*, surprise [24], [26], [54]).

Furthermore, discrete emotion effects in single word processing should not be specific to lexical decision but generalize to other word recognition tasks. If contextual learning is the basis of the discrete emotion effects discussed here, we would predict similar effects in naming and recognition memory performance for single words. Studies in the context of discrete emotion influences on attention (*e.g.*, in the emotional Stroop task, see [55]) may be of special interest, too. Shifted attention is commonly used to denominate effects of negative valence in word processing (*e.g.*,[56]), and different attention demands across the discrete emotion categories could bridge word processing and the underlying neural systems for discrete emotions.
